# Characterization of Host Cell Potential Proteins Interacting with OsHV-1 Membrane Proteins

**DOI:** 10.3390/v13122518

**Published:** 2021-12-15

**Authors:** Jiangnan Yu, Ying Liu, Bowen Huang, Chen Li, Dandan Wang, Mengli Yao, Lusheng Xin, Changming Bai, Chongming Wang

**Affiliations:** 1 Qingdao Key Laboratory of Mariculture Epidemiology and Biosecurity, Key Laboratory of Maricultural Organism Disease Control, Ministry of Agriculture, Yellow Sea Fisheries Research Institute, Chinese Academy of Fishery Sciences, Qingdao 266071, China; yujiangnan1996@outlook.com (J.Y.); huangbwn87@outlook.com (B.H.); lichen@ysfri.ac.cn (C.L.); wdd-wd@hotmail.com (D.W.); menglili1129@outlook.com (M.Y.); 2 Function Laboratory for Marine Fisheries Science and Food Production Processes, Qingdao National Laboratory for Marine Science and Technology, Qingdao 266071, China; 3 Key Laboratory of Environment Controlled Aquaculture, Dalian Ocean University, Ministry of Education, Dalian 116023, China; yingliu@dlou.edu.cn

**Keywords:** ostreid herpesvirus 1, membrane proteins, interaction, hemocytes, pull-down

## Abstract

The interaction between viral membrane associate proteins and host cellular surface molecules should facilitate the attachment and entry of OsHV-1 into host cells. Thus, blocking the putative membrane proteins ORF25 and ORF72 of OsHV-1 with antibodies that have previously been reported to subdue OsHV-1 replication in host cells, especially ORF25. In this study, prey proteins in host hemocytes were screened by pull-down assay with recombinant baits ORF25 and ORF72, respectively. Gene Ontology (GO) analysis of these prey proteins revealed that most of them were mainly associated with binding, structural molecule activity and transport activity in the molecular function category. The protein–protein interaction (PPI) network of the prey proteins was constructed by STRING and clustered via K-means. For both ORF25 and ORF72, three clusters of these prey proteins were distinguished that were mainly associated with cytoskeleton assembly, energy metabolism and nucleic acid processing. ORF25 tended to function in synergy with actins, while ORF72 functioned mainly with tubulins. The above results suggest that these two putative membrane proteins, ORF25 and ORF72, might serve a role in the transport of viral particles with the aid of a cytoskeleton inside cells.

## 1. Introduction

Herpesviruses can infect a broad range of hosts both on land and in the sea, and they have evolved successful approaches to infect different cell types, starting with virus attachment and entry into target cells [1]. In contrast to small enveloped viruses that encode one or two membrane glycoproteins to mediate their entry into specific cells, herpesviruses are equipped with more than a dozen membrane glycoproteins, which endows them with more opportunities and options when ascertaining successful entry according to cell types [2]. Some of the interactions between herpesvirus glycoproteins and cellular membrane molecules are believed to concentrate virions on the target cellular membranes only, such as the charge-based binding between glycoproteins (mainly gC and gB in the case of alpha-herpesviruses) and heparan sulfate. Conversely, other interactions are believed to trigger membrane fusion and virus entry [3]. In the majority of herpesviruses, the regulator heterodimer gH-gL and the viral fusion protein gB represent a core set of entry glycoproteins. The specific binding between glycoproteins and cell corresponding entry receptors triggers gB to execute membrane fusion, such as the binding between gD of HSV-1 (herpes simplex viruse-1) and host cellular membrane nectin-1 [4], and the binding between gp42 of EBV (Epstein–Barr virus) and human leukocyte antigen (HLA) class II molecules on B lymphocytes [5].

Alongside direct viral envelope fusion with the host cell membrane, herpesviruses can also adopt cell-to-cell spread, pH-dependent and pH-independent endocytic entry routes according to target cell types [6]. Cell-to-cell spread of herpesviruses mainly occurs in polarized cells, including epithelial cells and neuronal cells. Herpesviruses use cell junction adhesion proteins as their binding receptors via cell-to-cell spread; this route also helps herpesviruses to avoid being detected by the host immune system [7]. The core entry set of glycoproteins (gB, gH-gL) and gD receptors, as well as gK, play important roles in cell-to-cell spread [8]. Via the pH-dependent endocytic pathway, the virions are internalized and transported into the host cellular early endosomes. The mild acidic pH of the endosome triggers conformational changes in gB to fuse the viral envelope with the vesicular membrane [9]. However, the pH condition does not seem necessary for all of the endocytic herpesviruses entries. The low acidic pH is not required for the fusion of the HSV envelope with the endosomal membrane in C10 murine melanoma cells [10]. In addition, duck HBV (hepatitis B virus) enters cells via a low-pH-independent endocytic pathway, and EBV can enter cells via an endocytic route that is not affected by ammonium chloride [11,12]. The low-pH-independent endocytic routes may be explained by the occurrence of membrane fusion prior to acidification [10]. Once access to the cytosol is achieved, virions travel toward the nucleus along the cytoskeleton in the cytoplasm. Microtubules facilitate the long-distance transport of virions, while actin filaments facilitate short-range movements, especially in areas with a high ratio of actin to microtubule density. Virions migrate to and dock at nuclear pores, where they subsequently transfer their genome into the nuclear pore [13].

Herpesviruses are genetically classified into three families: Herpesviridae, Alloherpesviridae and Malacoherpesviridae [14]. Ostreid herpesvirus 1 (OsHV-1), as a lethal epidemic pathogen among bivalves, belongs to the family of Malacoherpesviridae. Massive global mortality events of cultures of bivalves frequently occurred following OsHV-1 infection. In particular, OsHV-1 infection is expounded to be the trigger of Pacific Oyster Mortality Syndrome (POMS), which has plagued the oyster industry for years [15]. As a member of the herpesvirus, the phenotype of OsHV-1 virion resembles that of other herpesviruses. OsHV-1 virion is spherical and comprises four major components: the core, the capsid, the tegument and the envelope. The core consists of a linear double-stranded DNA molecule. The capsid encapsulates the viral genetics, forming an icosahedron with an external diameter of 125–130 nm. The tegument protein layer surrounds the capsid. The outermost layer is the lipid envelope embedded with glycoproteins. According to the sequenced genomes of OsHV-1 or its variants, a total of 15 genes encode proteins that contain predicted signal or transmembrane domains and may contribute to the entry step of OsHV-1 or its variants [16,17,18].

The functions of the OsHV-1 genome encoding proteins are largely unknown; thus, the mechanism of OsHV-1 entry into cells hangs in doubt. Most OsHV-1 genome encoding proteins do not share sequential homology with those in the available protein database [19], and there are no mature bivalve cell lines for modeling OsHV-1 infection, which hinders the research in this field [20]. ORF25, ORF41 and ORF72 are the three putative membrane proteins of OsHV-1 that were initially selected for functional evaluation at the individual level. The ORF25 protein appeared to be involved in the interaction between OsHV-1 and host cells even if the other two proteins were likely implicated. The viral transcript amount showed the most significant decrease in hemolymph that had been incubated with viral suspension blocked by anti-ORF25 antibodies, followed by anti-ORF72 and anti-ORF41 antibodies [21]. With the aim of further investigating the molecular interaction between OsHV-1 and the host and of clarifying how the putative membrane proteins of OsHV-1 are implicated in virus entry into cells, we have carried out the pull-down assay with baits ORF25 and ORF72 to screen potential associate proteins in the lysate of host hemocytes. The prey proteins were identified by MS/MS and annotated.

## 2. Materials and Methods

### 2.1. Recombinant Expression and Purification

Based on the full-length DNA sequences of ORF25 and ORF72, two pairs of primers, ORF25F/ORF25R and ORF72F/ORF72R, were designed to amplify the ORF25 and ORF72 fragments, respectively (Table 1). For ORF25, the PCR product was digested with two restriction endonucleases, BamHI and NotI (FastDigest; Thermo Scientific, CA, USA). The obtained fragment and a pET30a plasmid digested with the same enzymes were ligated together using T4 DNA ligase to construct the recombinant plasmid pET30a-ORF25. The recombinant plasmid pET30a-ORF72 was also constructed as per the above description, with another two restriction endonucleases, EcoRI and NotI. Then the recombinant plasmid was transformed into competent *E. coli* transetta (DE3) (TransGen, Peking, China) for recombinant expression.

For the large-scale culture of bacteria, 6 mL of overnight-cultured *E. coli* transetta (DE3) was added to 300 mL of fresh LB liquid medium and cultured at 37 °C for 3~4 h (OD600 value reaching 0.4–0.6). Next, 0.2 mM isopropyl -D-1-thiogalactopyranoside (IPTG) was added to the LB medium (final concentration of 0.2 mM) to induce protein expression, and the bacteria were cultured at 37 °C for another 6 h.

These two recombinant proteins, rORF25 and rORF72, were largely expressed in inclusion bodies. The recombinant proteins were purified by a Ni2+ chelating Sepharose column under denatured conditions, with a final elution using 250 mmol L^−1^ imidazole (8 mol L^−1^ urea). The purified proteins were renatured for 12 h under a series of gradient urea-TBS glycerol buffers (50 mmol L^−1^ Tris–HCl, 50 mmol L^−1^ NaCl, 15% glycerol, 2 mmol L^−1^ reduced glutathione, 0.2 mmol L^−1^ oxide glutathione, a gradient urea concentration of 6, 4, 3, 2, 1, 0 mol L^−1^, pH 8.0) at 4 °C.

### 2.2. Pull-Down Assays

To explore the potential interaction molecules with ORF25 and ORF72, pull-down assays were performed. Hemocytes from ark clams were collected and lysed with cell lysis buffers (20 mM Tris pH 7.5, 150 mM NaCl, 1% Triton X-100, 1 mM phenylmethanesulfonyl fluoride). The supernatants were collected as homogenates after centrifugation for the following assays. An amount of 100 μg of recombinant ORF25 and ORF72 was incubated, respectively, with 50 μL Ni-Sepharose beads for 1 h. After the beads were washed with TBS three times, they were blocked with 5% BSA. After the blocked beads were washed with TBS another three times, they were incubated with prepared supernatants for 3 h. After the obtained beads were washed with 10 mM imidazole three times, they were boiled with 1× SDS protein Loading Buffer. Then, after transient centrifugation, the supernatants were analyzed by SDS-PAGE. The pull-down protocol using free Ni-Sepharose beads without incubation, with ORF25 or ORF72 in hemocytes, was also parallelly performed as a control. The prey proteins that were pulled down using ORF25 or ORF72 were identified by MS/MS using the Q-Exactive HF X mass spectrometer (Thermo Fisher Scientific, San Jose, CA, USA).

### 2.3. Protein Identification

Raw experimental MS data were converted into a peak list, and then matches were searched for in the theoretical MS/MS database using Mascot 2.3.02. software (http://www.matrixscience.com/, accessed on 12 August 2021). The search results were subject to strict filtering and quality control by FDR at spectral level (PSM-level FDR ≤ 0.01) to obtain a significant identified spectrum and peptide list. Gene ontology (GO) analysis was performed. Dor the GO entries involved in the three ontologies (cellular component, biological process, molecular function), a statistical chart was made according to the IDs and the number of all the corresponding proteins; the GO entries without the corresponding proteins were excluded.

### 2.4. Protein–Protein Interactions (PPI) Network

The potential pathways among prey molecules were indicated by the PPI network. The PPI network was built by the online STRING database (https://string-db.org/, version 11.5, accessed on 12 August 2021). The interactions (the default threshold > 0.4 in the STRING database) were selected to create the PPI network and further clustered by the K-means method.

## 3. Results

### 3.1. The Prey Proteins of the Recombinant ORF25 and ORF72 in Hemocytes

**Figure 1:**
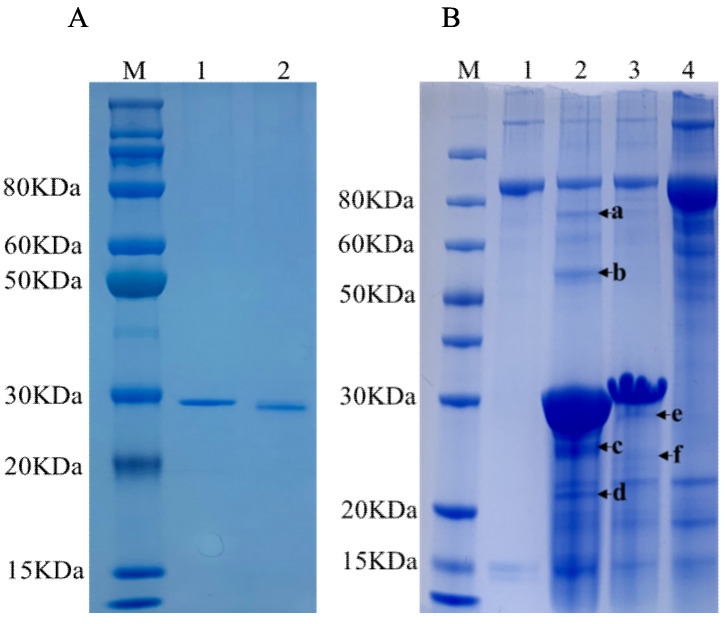
Pull-down assay of rORF25 and rORF72 in hemocyte lysates. (**A**) SDS-PAGE analyses of rORF25 and rORF72. Lane M: protein molecular standard (kDa); lane 1, purified rORF25; lane 2, purified rORF72. (**B**) rORF25 and rORF72 were used, respectively, to pull down interacting proteins in the lysates of hemocytes from ark clams, and the proteins were analyzed by SDS-PAGE. Free Ni2+ Sepharose beads were set as the control. Lane M, protein marker; lane 1, free Ni2+ Sepharose beads; lane 2, Ni2+ Sepharose beads binding with rORF72; lane 3, Ni2+ Sepharose beads binding with rORF25; lane 4, the input of hemocyte lysates, a–f represents specific bands in lane 2 and 3.

The purified recombinant ORF25 and ORF72 were analyzed by SDS-PAGE. Two distinct bands with a molecular mass of 30 kDa and 25 kDa were revealed, respectively, which was consistent with their predicted molecular mass (Figure 1A).

To further elucidate the roles of ORF25 and ORF72 in the interaction between OsHV-1 and its hosts, we first used rORF25 and rORF72, respectively, to pull down potential interacting proteins in the hemocytes of ark clams. We obtained two specific bands via SDS-PAGE analysis in the rORF25 pull-down solution compared to the control (free Ni2+ Sepharose beads) pulldown solution, and we obtained four in the rORF72 pulldown solution (Figure 1B). The bands were identified by tandem mass spectrometry (MS/MS). A total of 45 spectra were matched and 36 proteins identified from band a (Supplementary Table S1). A total of 1347 spectra were matched and 326 proteins identified from band b (Supplementary Table S2). A total of 96 spectra were matched and 55 proteins identified from band c (Supplementary Table S3). A total of 224 spectra were matched and 56 proteins identified from band d (Supplementary Table S4). A total of 81 spectra were matched and 59 proteins identified from band e (Supplementary Table S5). A total of 68 spectra were matched and 48 proteins were identified from band f (Supplementary Table S6).

### 3.2. The Functional Annotation of Prey Proteins

**Figure 2:**
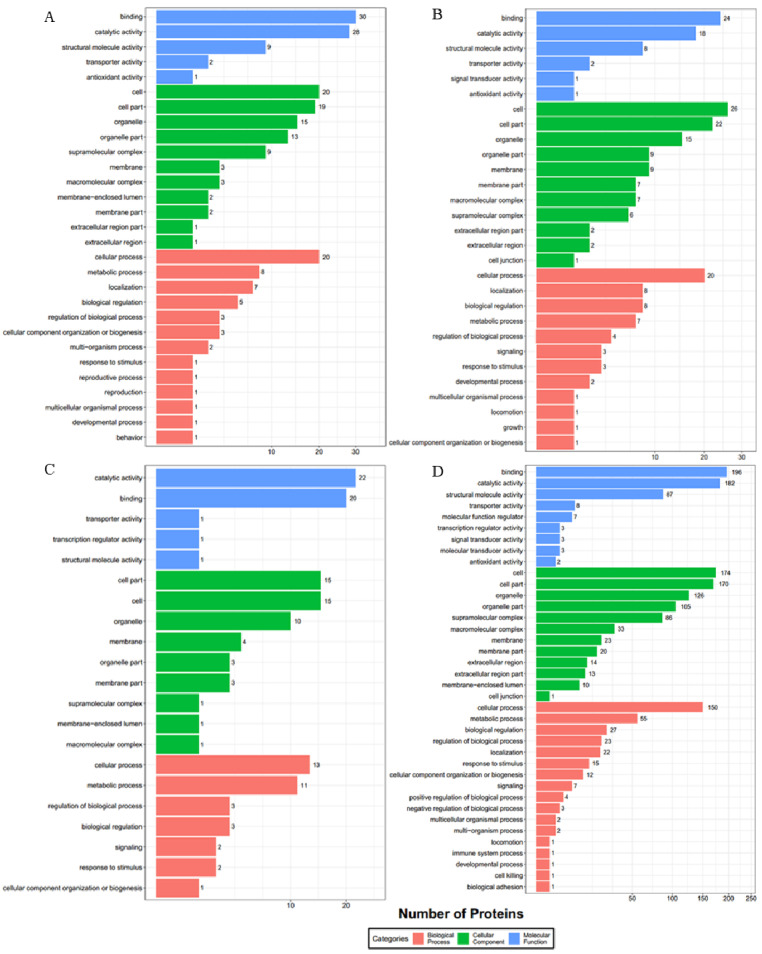
Prey proteins’ distribution by GO at level 2 for bait ORF72 (Red: Biological process; Blue: Molecular function; Green: Cellular component). Numbers shown in the chart represent the number of prey proteins with corresponding GO terms. Subfigures (**A**–**D**) correspond to the results of GO analyses for the identified proteins in bands a, b, c and d.

The GO functional analysis of the prey proteins for bait ORF72 was applied. A total of 36 prey proteins from band a, 326 prey proteins from band b, 55 prey proteins from band c and 56 prey proteins from band d were assigned with GO terms (level-two), which were summarized into three main categories: biological process, molecular function and cellular component (Figure 2). In the molecular function category, the top three GO terms were binding (GO: 0005488), catalytic activity (GO: 0003824) and structural molecule activity (GO: 0005198) for band b, c and d. Conversely, the top three GO terms were catalytic activity (GO: 0003824), binding (GO: 0005488) and transporter activity (GO: 0005215) for band a (Figure 2A). In the biology process category, cellular process (GO: 0009987) accounted for the majority for all bands. The cellular component category mainly consisted of the cell (GO: 0005623) and cell part (GO: 0044464), then organelle (GO: 0043226), membrane (GO: 0016020) and others for all bands.

**Figure 3:**
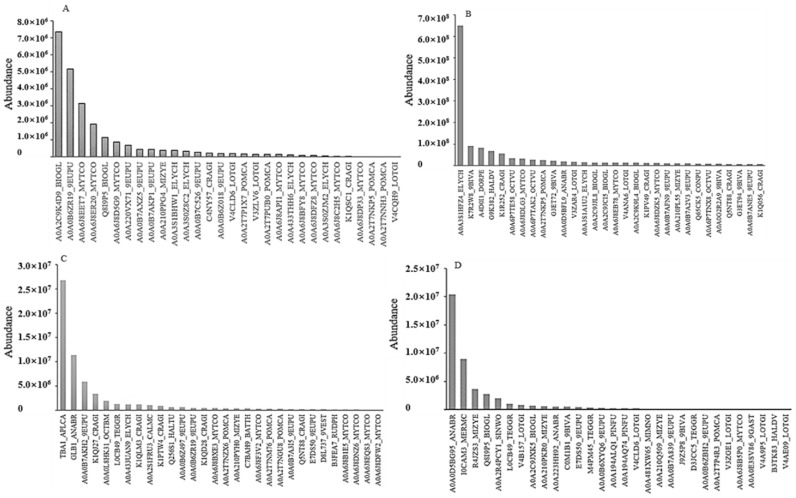
Top 30 most abundant prey proteins for bait ORF72 by MS/MS analysis. Subfigures (**A**–**D**) correspond to the results for the proteins identified in bands a, b, c and d.

For bait ORF72 (Figure 3), the three most abundant prey proteins were two 1,4-alpha-glucan branching enzymes (protein ID: A0A2C9K4D9_BIOGL, A0A0B6ZR19_9EUPU) and a tyrosine-protein phosphatase (protein ID: A0A6J8EET7_MYTCO) that were identified in band a by MS/MS. An ATP-binding cassette (ABC) transmembrane type-1 domain-containing protein (protein ID: A0A6J8EET7_MYTCO) was also identified in band a. Tubulin subunits (alpha chain protein ID: A0A3S1HFZ4_ELYCH, K7R2W8_9BIVA; beta chain protein ID: A4D0I1_DORPE, G9K382_HALDV) took the top four spots of abundance in band b, a galectin (protein ID: A0A0D5BFL9_ANABR) was also found in band b. Tubulin alpha-1 chain (protein ID: TBA1_APLCA), globin-1(protein ID: GLB1_ANABR) and an uncharacterized protein (protein ID: A0A0B7AKH2_9EUPU) were the highest three in band c. Ferritin (protein ID: A0A0D5BG95_ANABR, I0CAM3_MERMC and R4JZS3_MIZYE) accounted for the majority in band d. A galectin (protein ID: M4PM45_TEGGR) and a calmodulin (protein ID: A0A210PKR0_MIZYE) were found together in band d.

**Figure 4:**
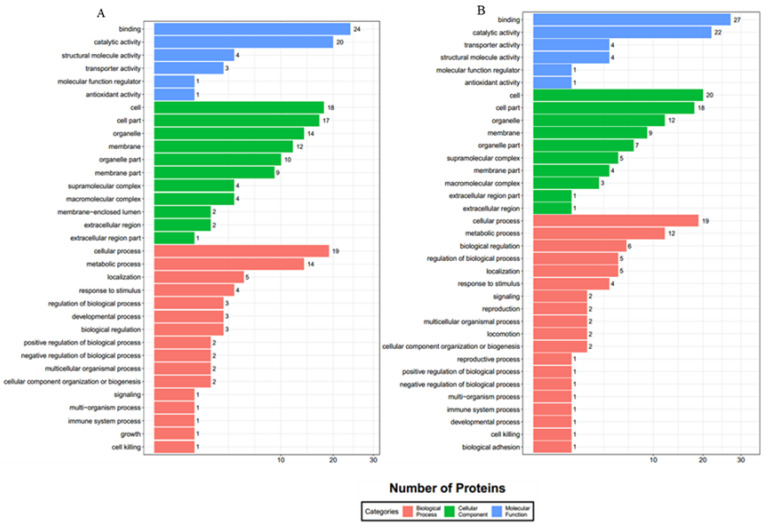
Prey proteins’ distribution by GO at level 2 for bait ORF25 (Red: Biological process; Blue: Molecular function; Green: Cellular component). Numbers shown in the chart represent the number of prey proteins with corresponding GO terms. Subfigures (**A**,**B**) correspond to the results of GO analyses for the identified proteins in bands e and f.

A total of 59 prey proteins from band e and 48 prey proteins from band f were assigned with GO terms (level-two) for bait ORF25 (Figure 4). In the molecular function category, prey proteins were mainly clustered in binding (GO: 0005488), catalytic activity (GO: 0003824), structural molecule activity (GO: 0005198), transporter activity (GO: 0005215), molecular function regulator (GO: 0098722) and antioxidant activity (GO: 0016209) for band e and f. In the biology process category, cellular process (GO: 0009987) and metabolic process (GO: 0008152) accounted for the majority of these two bands. The cellular component category mainly consisted of the cell (GO: 0005623) and cell part (GO: 0044464) and organelle (GO: 0043226) for these two bands.

**Figure 5:**
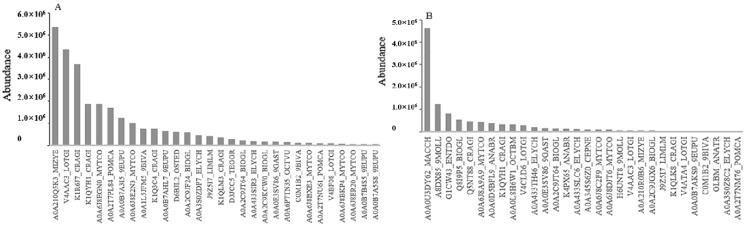
Top 30 most abundant prey proteins for bait ORF25 by MS/MS analysis. Subfigures (**A**,**B**) correspond to the results for the proteins identified in bands e and f.

For bait ORF25 (Figure 5), a urease accessory protein (protein ID: A0A210Q5K3_MIZYE), an alpha-amylase (protein ID: V4AAC3_LOTGI) and an actin (protein ID: K1R6J7_CRAGI) were revealed as the three most abundant proteins in band e by MS/MS. a thioredoxin peroxidase (protein ID: A0A0U3DY62_MACCH), a glyceraldehyde-3-phosphate dehydrogenase (protein ID: A8DX85_9MOLL) and an elongation factor 1-alpha (protein ID: G1CW43_ENTDO) were the most abundant proteins in band f. A galectin (protein ID: A0A210PKR0_MIZYE) was also found in band f.

### 3.3. The PPI of Prey Proteins

**Figure 6:**
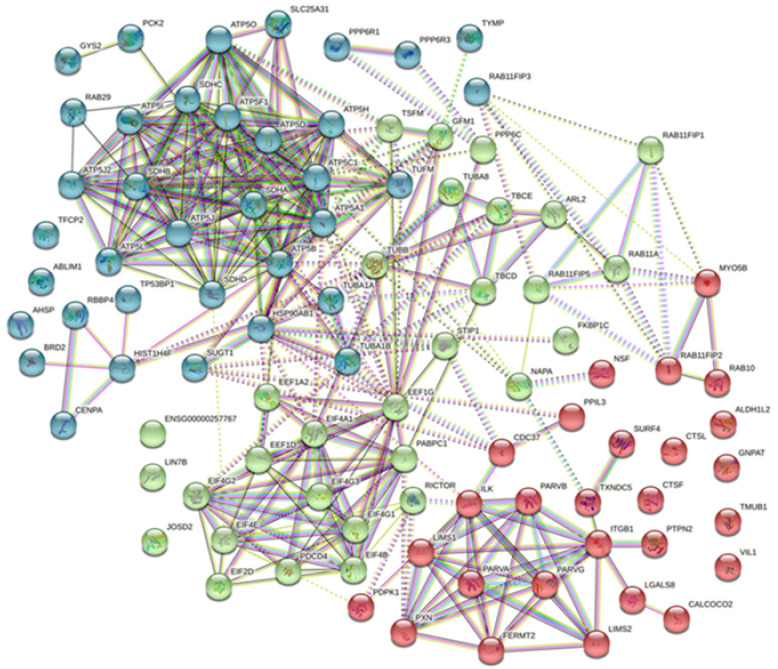
Protein–protein interaction (PPI) maps of prey proteins for bait ORF72. Network nodes represent proteins. Edges represent protein–protein associations, edges between clusters are dotted lines. Different colors represent three clusters that were distinguished by the K-means method, and the gene name information was listed in Supplementary Table S7.

To evaluate the relationship among all prey proteins by pull-down assay for baits ORF72 and ORF25, respectively, we performed STRING analysis to construct protein–protein interaction networks. Most of the prey proteins were interlinked. For bait ORF72 (Figure 6), the interactions of prey proteins mainly included cytoskeleton assembly (with hub genes tubulins TUBB, TUBA1A, TUBA1B and TUBA8; tubulin folding cofactors TBCE and TBCD), energy metabolism (with hub genes ATP synthesis units ATP5A1, ATP5B, ATP5C1 and others; succinate dehydrogenase cytochrome subunits SDHA, SDHB, SDHC and SDHD), nucleic acid binding (with hub genes centromere protein CENPA, histone HIST1H4F, polyadenylate-binding protein PABPC1, RNA cap-binding EIF4E, EIF4G, EIF4A1 and others) and regulation of cell adhesion and cytoskeleton organization (with hub genes parvin units PARVA, PARVB and PARVG, adaptor proteins LIMS1 and LIMS2) (Figure 6).

**Figure 7:**
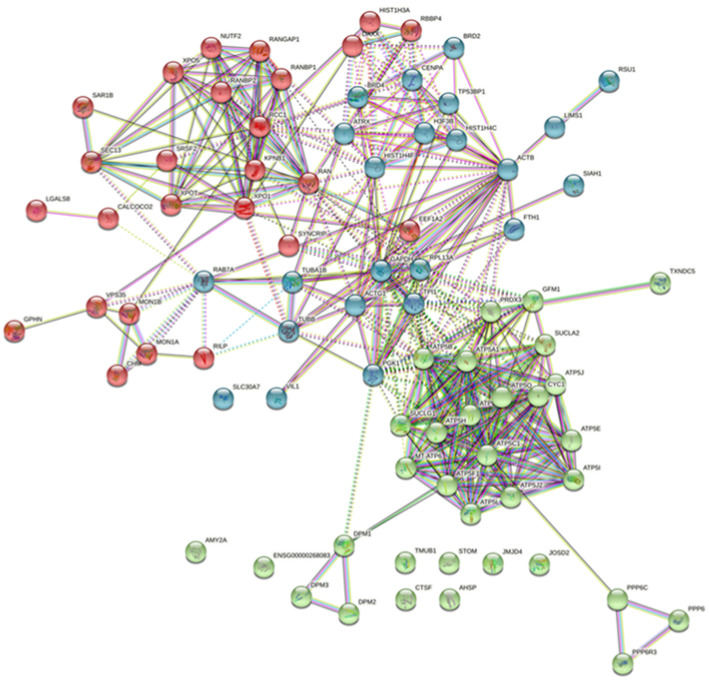
Protein–protein interaction (PPI) maps of prey proteins for bait ORF25. Network nodes represent proteins. Edges represent protein–protein associations, edges between clusters are dotted lines. Different colors represent three clusters that were distinguished by the K-means method, and the gene name information was listed in Supplementary Table S7.

For bait ORF25 (Figure 7), the interaction of the prey proteins mainly included energy metabolism (with hub genes ATP5A1, ATP5B, ATP5C1 and others), cytoskeleton assembly (with hub genes actins ACTB and ACTG1; tubulins TUBB and TUBA1B), nuclear transport-associated proteins (with hub genes exportins XPOT, XPO1 and XPO5; GTP-binding nuclear protein RAN; regulator of chromosome condensation RCC1; nuclear transport factor NUTF2) (Figure 7).

## 4. Discussion

Though the morphological features of OsHV-1 resemble that of other herpesviruses, the genome sequence of OsHV-1 shares less homology with others, especially sequence domains encoding membrane proteins. Tens of putative membrane proteins are encoded in the OsHV-1 genome, which may endow OsHV-1 with multiple tissue tropism. OsHV-1 particles had been observed in the mantle cells and hemocytes of infected ark clams and oyster spats by transmission electron microscope or in situ hybridization [22,23]. There should be diverse entry receptors in host cells for OsHV-1 resembling the corresponding hosts of other herpesviruses. Three viral proteins (ORF25, ORF41 and ORF72) were initially selected to identify whether they were implicated in OsHV-1 intruding cells by the method of antibody blocking. The mortality rate of the spat decreased significantly in the group that had been inoculated with the viral suspension blocked with the three antibodies (anti-ORF25, anti-ORF41 and anti-ORF72) in comparison to the group inoculated solely with viral suspension. Significantly lower viral transcripts were also observed in the hemolymph that had been incubated with the viral suspension containing three viral proteins or their corresponding antibodies, compared to the hemolymph that had been incubated solely with viral suspension. Thus, it was thought that the three viral proteins, mainly the protein encoded by ORF25, might be implicated in the virus invading oyster cells [21]. In this study, further investigation into how host cell proteins interacted with ORF25 and ORF72 was carried out by pull-down assay, revealing how these two viral proteins might function in an OsHV-1 infection.

The majority of the captured prey proteins for both baits ORF25 and ORF72 were clustered into the binding term in the molecular function category by GO. The binding term consisted of abundant cytoskeleton assembly associated proteins for both baits. Due to the sticky cytoplasm, there is a formidable barrier for the free diffusion of virions to nuclei. The cytoskeleton is commonly usurped by herpesviruses, as it facilitates highly efficient transport through cytoplasm. Microtubules are essential for long-distance transport, while actin filaments function in short-range movements [13,24]. ORF25 and ORF72 might serve in the OsHV-1 transport in synergy with the cytoskeleton. Actins were principally found paired with ORF25, were in high abundance and served as the hub genes in the prey proteins of ORF25, while tubulins were principally paired with ORF72. The transport dependent on cytoskeletons consumes energy. There was a high abundance of energy metabolism associated proteins in the prey proteins of ORF25 and ORF72, which might contribute towards the energy requirement of the synergistic effect between these two viral proteins and cytoskeletons. Alongside intracellular transport, actins are also implicated in the endocytosis of extracellular virions [25]. Actins are required for HSV-1 to adopt the pH-dependent endocytic pathway to enter corneal fibroblasts (CF) and CHO cells because this process was proved to be inhibited by cytochalasin D [26]. In actin-dependent entry, virions trigger the rearrangement of actins and the formation of protrusions; virions move along these protrusions for eventual endocytosis [27]. The synergistic effect between ORF25 and actins might be blocked by antibodies against ORF25, which inhibit the entry and transport process of OsHV-1, which has resulted in the decreased load of OsHV-1 in host cells in previous studies.

The ABC transmembrane type-1 domain-containing protein was traced in the prey proteins of ORF72. Eukaryotic ABC transporters mainly function by expelling solutes from the cytosol out of the cell or into subcellular organelles, such as the lumen of the ER, lysosomes and peroxisomes [28]. ORF72 might target this protein along the cytoskeleton and then serve in the mature and release process of OsHV-1. Alternatively, the ABC transporter might form the central part of the major histocompatibility complex class I (MHC I) peptide-loading machinery. ICP47 encoded by the herpes simplex virus (HSV 1 and 2) competes with peptides for the binding pocket on TAP, which prevents antigen translocation and MHC I loading in virally infected cells [29]. While it seems that the MHC I in mollusks does not mediate antigen processing, further research is required to explore whether the synergistic effect between ORF72 and host ABC transport-related genes would enable OsHV-1 to escape the immune monitor of mollusk hosts. Calmodulin was also found in the prey proteins of ORF72. Calmodulin, as an intracellular calcium-binding protein, regulates several key enzymes, some of which directly affect cytoskeletal elements, although they might also interact nonspecifically with other cellular constituents. Haloperidol and R24571, as calmodulin inhibitors, were found to be capable of blocking EBV infection [30]. Calmodulin might play a regulation role in the interaction between ORF72 and the cytoskeleton. The transcripts of ORF72 were found early, just two hours after the OsHV-1 infection [31], which further indicated that ORF72 might function at the early stage of the virion maturation and transport process.

Galectins could be found in the prey proteins of both ORF25 and ORF72. They constitute a lectin family with an affinity for β-galactosides and a wide taxonomic distribution. As pathogen model molecular receptors (PRRs), galectins have been discovered to bind glycans on the surface of viruses, bacteria, protista and fungi [32]. Galectin-1 can inhibit virus attachment and host cell fusion by binding N-linked oligosaccharides from the viral glycoproteins and promoting their crosslinking and oligomerization. A paramyxovirus, Nipah virus, can invade the host endothelial cells by attachment and fusion of its envelope glycoproteins. While the dimeric galectin-1 specifically crosslinks the N glycans displayed in the envelope glycoproteins, hampering the interaction between glycoproteins and their corresponding receptors and blocking membrane fusion [33]. Vertebrate galectins are known to participate at various levels of antiviral defense, from the initial recognition and blocking of fusion glycoproteins to the motivation of the host immune system [34]. However, the understanding of whether the interaction between mollusk galectins and OsHV-1 glycoproteins facilitates or inhibits OsHV-1 replication still needs to be established.

## 5. Conclusions

In conclusion, host prey proteins associated with baits ORF25 and ORF72 were screened in the hemocyte lysate by pull-down assay, respectively. Hundreds of prey proteins were traced for both baits. Gene Ontology (GO) analysis of these prey proteins revealed that most of them were mainly associated with binding, transport activity and structural molecule activity in the molecular function category. For both ORF25 and ORF72, abundant cytoskeleton assembly elements were identified among prey proteins, together with regulators of cytoskeleton and energy metabolism. Abundant actins could be found in the prey proteins of ORF25, while tubulins could be found in those of ORF72. The above results suggested that these two putative envelop proteins, ORF25 and ORF72, might serve a role in the transport of virions with the aid of cytoskeleton inside cells. ORF25 tended to function in synergy with actins, while ORF72 mainly functioned with tubulins. Some other important potential molecules were identified that could synergize with ORF72 and ORF25, such as the ABC transmembrane type-1 domain-containing protein, calmodulin and galectin. However, this study was unable to discover whether these prey proteins interact directly or indirectly with these two baits, and these interactions need to be further investigated. Overall, our research refreshed the functional cognitions of the two putative membrane proteins of OsHV-1, aiding further functional studies on these two putative membrane proteins.

## Figures and Tables

**Table 1:** Primers used in this study.

Primer Name	Sequence (5′-3′)
Fragments amplifying primers	
ORF25F	ATGACTTTAGCTGCTAAGTTAATAGT
ORF25R	CTAATGTAAATATACCCTTCTCAG
ORF72F	ATGGCAACAGACCAACAAGACC
ORF72R	TTACTTAAAGAGGTCTTCATAATTC
ORF primers for prokaryotic expression	
ORF25ES	CGCGGATCCATGACTTTAGCTGCTAAGTTAATAG
ORF25EA	AAGGAAAAAAGCGGCCGCAATGTAAATATACCCTTCTCAG
ORF72ES	CGCGAATTCATGGCAACAGACCAACAAGAC
ORF72EA	AAGGAAAAAAGCGGCCGCACTTAAAGAGGTCTTCATAATTC

## Supplementary Materials

The following are available online at https://www.mdpi.com/article/10.3390/v13122518/s1, Tables S1–S6: protein identification list in band a, b, c, d, e, f; Table S7: abbreviation in the PPI.
